# MULTIPLE CHRONIC CONDITIONS AND ACCELERATED AGING IN PEOPLE EXPERIENCING HOMELESSNESS

**DOI:** 10.1093/geroni/igz038.2885

**Published:** 2019-11-08

**Authors:** Julie Zuniga, Whitney Thurman, Dong Eun Jang

**Affiliations:** 2 University of Texas at Austin College of Pharmacy, Austin, Texas, United States; 1 The University of Texas at Austin School of Nursing, Austin, Texas, United States

## Abstract

The median age of the U.S. homeless population is increasing, and PEH often experience accelerated aging compared to the general population. Moreover, PEH have disproportionately high rates of chronic illness, psychological distress, and poor health status. The purpose of this study was to investigate associations between morbidity, psychological distress, and healthcare utilization in PEH. The specific aims of this study were to: 1) determine the prevalence of multiple chronic conditions in PEH, 2) analyze whether prevalence varies by level of psychological distress, and 3) analyze associations between age, health conditions, and psychological distress. We used data from the 2010 National Health Interview Survey. Analysis included descriptive statistics, t-tests, chi-square tests, ANOVA, and correlations. Homelessness was operationalized using the variable from the NHIS asking if the individual had spent >24 hours homeless in the past year. Measures included demographics, 11 chronic conditions, and psychological distress. PEH (n = 1809) were majority male (68.9%), White (66%), and unmarried (61.1%). Mean age of PEH was significantly higher than housed individuals (43.5 vs. 38.4 years, p < .000). Chi-square tests indicated that the prevalence of hepatitis, heart disease, emphysema, arthritis, epilepsy, and asthma were significantly higher (p < .01) in the PEH group than in the general population. Psychological distress differed significantly by morbidity. The relatively young mean age and the high burden of morbidity suggest that aging may be progressing more rapidly in PEH than previously recognized. Future research is needed to investigate the trajectory of aging in this population.

